# A humanized NOG‐EXL mouse model for producing severe fever with thrombocytopenia syndrome virus–reactive human antibodies

**DOI:** 10.1002/ame2.70140

**Published:** 2026-02-26

**Authors:** Dong Hoon Lee, Jiyeong Bae, Sumi Kim, Chan Young Song, Jung Hyu Shin, Eun Hee Kim, Chan Ho Jang, Young‐sun Yun, Dong‐sook Lee, Hyuk Chu, Jang‐Hoon Choi, Chan Woo Kim

**Affiliations:** ^1^ Division of Acute Viral Diseases Center for Emerging Virus Research, National Institute of Infectious Diseases, National Institute of Health Cheongju Korea; ^2^ Experimental Evaluation Department Non‐Clinical Evaluation Center, OSONG Medical Innovation Foundation (KBIOHealth) Cheongju Korea; ^3^ College of Pharmacy and Medical Research Center Chungbuk National University Cheongju Korea

**Keywords:** human immunoglobulin G (IgG), humanized mouse, severe fever with thrombocytopenia syndrome virus (SFTSV)

## Abstract

**Background:**

Humanized mouse models are essential for studying the human immune response and antibody development. However, conventional models show limited B cell maturation and antigen‐specific humoral responses. To overcome these limitations, we used the NOG‐EXL mice expressing human interleukin 3 (IL‐3) and granulocyte‐macrophage colony‐stimulating factor (GM‐CSF) to enhance myeloid and B‐cell lineage differentiation.

**Methods:**

Human CD34^+^ hematopoietic stem cells (HSC) were transplanted into NOG‐EXL mice to produce humanized immune systems. After immune cell reconstitution was confirmed across 12 weeks, the mice were immunized twice with inactivated severe fever with thrombocytopenia syndrome virus (SFTSV) antigens. Peripheral blood mononuclear cells and splenocytes were analyzed using multicolor flow cytometry to assess human immune cell subsets. Antigen‐specific immunoglobulin G (IgG) production was quantified using enzyme‐linked immunosorbent assay (ELISA), and virus‐specific B cells were isolated using antigen‐labeled recombinant protein probes.

**Results:**

Twelve weeks after transplantation of HSCs into NOG‐EXL mice, they exhibited robust engraftment of human leukocytes, including T, B, and dendritic cells, compared to NOG mice. Unlike NOG mice, humanized NOG‐EXL mice exhibited an increase in human IgG levels, indicating the production of human antibody responses to antigens. Humanized NOG‐EXL mice were immunized twice every 2 weeks with inactivated SFTSV, and antigen‐specific human antibodies against the virus were detected in the mouse sera by ELISA. Sera from SFTSV‐immunized humanized mice demonstrated neutralizing activity against SFTSV, confirming the induction of functional virus‐specific neutralizing antibodies. Antigen‐binding IgG‐positive human B cells were isolated from mouse splenocytes using recombinant protein probes.

**Conclusion:**

This model provides a valuable platform for evaluating humoral immunity and isolating B cells using high‐affinity human monoclonal antibodies without genetic engineering.

## Graphical abstract

Humanized mouse models are essential for studying human immune response and antibody development. However, conventional models exhibit limited B cell maturation and antigen‐specific humoral responses. To overcome these limitations, we used the NOG‐EXL mice expressing human interleukin 3 (IL‐3) and granulocyte‐macrophage colony‐stimulating factor (GM‐CSF) to enhance myeloid and B cell lineage differentiation. Human CD34^+^ hematopoietic stem cells (HSC) were transplanted into NOG‐EXL mice to produce humanized immune systems. After immune cell reconstitution was confirmed across 12 weeks, the humanized mice were immunized twice with inactivated severe fever with thrombocytopenia syndrome virus (SFTSV) antigens. Peripheral blood and spleen were analyzed using multicolor flow cytometry to assess human immune cell subsets. Antigen‐specific immunoglobulin G (IgG) production was quantified using enzyme‐linked immunosorbent assay (ELISA), and virus‐specific B cells were isolated using antigen‐labeled recombinant protein probes. Twelve weeks after transplantation of HSCs into NOG‐EXL mice, they exhibited robust engraftment of human leukocytes, including T, B, and dendritic cells, compared to NOG mice. Unlike NOG mice, humanized NOG‐EXL mice exhibited an increase in human IgG levels, indicating the production of human antibody responses to antigens. The humanized mice were immunized twice every 2 weeks with inactivated SFTSV, and antigen‐specific human antibodies against the virus were detected in mouse sera using ELISA. Sera from SFTSV‐immunized humanized mice demonstrated neutralizing activity against SFTSV, confirming the induction of functional virus‐specific neutralizing antibodies. Antigen‐binding IgG‐positive human B cells were isolated from mouse splenocytes using recombinant protein probes. This model provides a valuable platform for evaluating humoral immunity and isolating B cells using high‐affinity human monoclonal antibodies without genetic engineering.

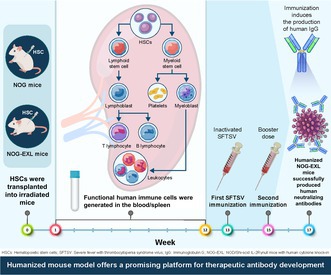

## 1 INTRODUCTION

Treating infectious diseases requires the development of effective therapeutic and prophylactic strategies. Of these, antibody‐based approaches are essential for detecting and treating viral infections.^1^, ^2^ Particularly, monoclonal human antibodies are used for their potent neutralizing activity against viruses, such as severe acute respiratory syndrome coronavirus 2 (SARS‐CoV‐2),^1^ reducing severe symptoms and hospitalization rates.^2^, ^3^ Thus, they are powerful tools for treating and preventing infectious diseases because of their high specificity and efficacy. However, producing fully human antibodies suitable for clinical applications requires samples from patients with infectious diseases or advanced experimental models that accurately mimic human immune responses.

Humanized mouse models, which involve engrafting various types of human cells and tissues, have advanced the understanding of human disease mechanisms and innovative treatment strategies.^4^ Humanized mouse models are immunodeficient mice with human immune cells.^5^, ^6^, ^7^, ^8^ These models can be produced through (1) inoculation with PBMCs (Hu‐PBMC) or (2) CD34^+^ hematopoietic stem cells (Hu‐HSC) and (3) engraftment with human fetal liver and thymic tissue (bone marrow‐liver‐thymus [BLT]) into the subrenal capsules of immunocompromised mice. The Hu‐PBMC model enables rapid assessment of human immune function because the transferred lymphocytes (most being CD3^+^ T cells) are functionally mature.^7^ Hu‐HSC models have a major human lymphoid lineage, including T, B, and partially developed innate immune cells, such as NK cells, monocytes, myeloid dendritic cells (mDC), and plasmacytoid dendritic cells (pDC). Fetal human HSCs engraft in the mouse bone marrow and allow progenitor cells to populate the mouse cells with human lymphoid and myeloid cell compartments. The transplanted human thymus tissue actively supports human T cells during the 13–18 weeks required for human immune reconstitution in mice; however, the procedure is complex and expensive.^5^, ^6^, ^7^

HSC‐transplanted humanized mice produce human monoclonal immunoglobulin M (IgM) antibodies against two commercial vaccine antigens: tetanus toxoid and hepatitis B surface antigen. This approach offers a proof of concept for producing human antibodies without genetic modifications.^8^ Similarly, humanized tumor mice, produced by co‐transplanting human stem and breast cancer cells into NSG mice, developed a human immune system and produced tumor‐specific antibodies, enabling full human monoclonal antibody production.^9^ Human interleukin 6 (IL‐6) knock‐in mice were produced to enhance adaptive immune responses in HSC‐transplanted humanized models. These mice exhibited improved T cell engraftment, increased memory B cells, and robust antigen‐specific immunoglobulin G (IgG) production, providing a novel tool for studying human immunity and vaccine strategies.^10^

HSC‐transplanted humanized mice are valuable tools for investigating human immune responses and developing human antibody therapies. After the introduction of human HSCs, murine models produce diverse human immune components, including lymphocytes, mononuclear phagocytes, antigen‐presenting cells, and cytotoxic lymphocytes.

The engraftment efficiency of human immune cell lineages in immunodeficient mice depends on the strain, prompting the development of various models to enhance the reconstitution of the humanized immune system. One of the highly immunodeficient transgenic (Tg) mice, NOD/Shi‐scid‐IL2rg null (NOG)‐EXL, expresses the human granulocyte‐macrophage colony‐stimulating factor (hGM‐CSF) and human interleukin 3 (hIL‐3), which stimulate the proliferation of hematopoietic cells and regulate inflammatory responses.^11^, ^12^, ^13^, ^14^ GM‐CSF regulates dendritic cells (DC) and macrophages crucial in T cell activation^11^ and antibody production by B cells. IL‐3 supports the survival and proliferation of T and B cells, indirectly promotes the differentiation of naive T cells into TH2 cells,^12^ stimulates IgG secretion from B cells,^13^ and promotes B‐cell proliferation and differentiation.^14^ In the development of humanized mice using NOG‐EXL mice, hIL‐3 and hGM‐CSF are essential for producing human antibodies by modulating immune cells. These cytokines enhance myeloid lineage expansion, influencing the B cell compartment. Their presence affects B‐cell maturation and function, impacting overall immune system reconstitution.^15^

In this study, we used the Tg strain NOG‐EXL mice to induce enhanced B cell development and antigen‐specific antibody responses. We selected a vector‐transmitted virus to confirm the production of virus‐specific human antibodies. Severe fever with thrombocytopenia syndrome (SFTS) is an emerging infectious disease in East Asian countries, particularly Korea, China, and Japan, where transmission occurs via tick vectors, such as *Haemaphysalis longicornis* and *Rhipicephalus microplus*. SFTS virus (SFTSV) contains three genomic segments—large (L), medium (M), and small (S)—and is a single‐stranded, negative‐sense RNA virus. The L segment encodes the RNA‐dependent RNA polymerase. The S segment encodes the nucleoprotein and the nonstructural protein NSs, with the nucleoprotein promoting RNA packaging. The M segment encodes the envelope glycoproteins Gn and Gc. On the virus surface, Gn and Gc form a heterodimer as the main antigenic unit, with Gn mediating receptor binding and serving as a major target for neutralizing antibodies, whereas Gc mediates membrane fusion during viral entry. Neutralizing antibody responses to both glycoproteins are actively studied.^16^, ^17^, ^18^, ^19^ In 2019, the International Committee on Taxonomy of Viruses designated SFTSV as Dabie bandavirus, and the virus has continued to spread with no available vaccines or treatments to date.^17^ Humanized mice based on the NOG‐EXL strain exhibited significantly high levels of virus‐specific human IgG after immunization with the inactivated virus. Binding antibodies against SFTSV were produced in the blood of humanized mice, and the production of neutralizing antibodies against SFTSV was confirmed. Furthermore, we isolated virus‐specific antibody‐producing B cells from the splenocytes of HSC‐transplanted humanized NOG‐EXL mice, establishing a platform for human antibody production.

## 2 METHODS

### 2.1 Animals and HSCs

NOD.Cg‐Prkdc^scid^Il2rg^tm1Sug^/ShiJic (NOG) male mice were purchased from Koatech Co., Ltd. (Republic of Korea), and NOD.Cg‐Prkdc^scid^Il2rg^tm1Sug^Tg(SRa‐IL3, SRa‐CSF2)/Jic (NOG‐EXL) male mice were purchased from In‐Vivo Science Inc. (Japan). Human CD34^+^ HSCs were obtained from Lonza (2C‐101).

### 2.2 HSC preparation

Cryopreserved CD34^+^ HSCs (2C‐101, Lonza) were rapidly thawed in a water bath at 37°C and resuspended in 9 mL of RPMI 1640 medium (11875093; Gibco) supplemented with 10% fetal bovine serum (FBS, 26140079; Gibco). The cell suspension was centrifuged at 1800 rpm for 10 min at 4°C, and the supernatant was carefully aspirated. The cell pellet was washed once with the same medium and centrifuged under the same conditions. After the supernatant was removed, the cells were resuspended in phosphate‐buffered saline (PBS, 700110044; Gibco). Overall, 5 × 10^4^ cells were prepared in 200 μL.

### 2.3 Production of humanized mice

All animal procedures were approved by the Institutional Animal Care and Use Committee. Six‐week‐old male NOG and 7‐week‐old male NOG‐EXL mice were subjected to total body X‐ray irradiation (M‐150WE, SOFTEX) at 1.5 Gy. Within 24 h, each mouse was intravenously transplanted with 5 × 10^4^ human CD34^+^ HSCs (2C‐101; Lonza). After transplantation, body weight was monitored weekly, and peripheral blood was collected from the orbital vein every 4 weeks to assess immune cell reconstitution via flow cytometry (BD FACSLyric). Peripheral blood was collected from humanized mice following immunization with inactivated virus. Red blood cells were lysed using 10× RBC lysis buffer (420301; BioLegend) diluted in distilled water (10977035; Gibco), and leukocytes were washed twice with FACS buffer (PBS containing 2% FBS). For in vivo immune profiling, multicolor flow cytometry was performed using fluorochrome‐conjugated antibodies. T cell subsets were identified with antihuman CD3–PE (555333), CD4–PE‐Cy7 (348799), and CD8–APC‐Cy7 (561945), and leukocytes were gated with CD45–fluorescein isothiocyanate (FITC) (560976) (all from BD Biosciences). B cells, activated lymphocytes, and NK cells were labeled using CD19–APC (555415), CD69–BV421 (562884), and CD56–BV510 (563041), respectively. Myeloid subsets were analyzed using CD11b–BV605 (663191), CD14–BV711 (563372), CD16–BV786 (563690), and CD11c–APC‐R700 (566875) (all BD Biosciences). Antibodies were incubated in 2% FBS buffer for 30 min on ice, protected from light. Data were obtained on a flow cytometer and analyzed using FlowJo software.

### 2.4 Analysis of human total IgG

Plasma was collected 2 weeks after the second subcutaneous immunization with 100 μg ovalbumin (OVA, 5821‐43‐02; InvivoGen) per mouse at 11 and 13 weeks posttransplantation. Total human IgG levels were measured using an enzyme‐linked immunosorbent assay (ELISA) kit (BMS2091; Invitrogen) following the manufacturer's instructions. Plates were coated with capture antibody, and standards, 10‐fold diluted NOG plasma, or 50‐fold diluted NOG‐EXL plasma were added and incubated. Detection used horseradish peroxidase (HRP)–conjugated antihuman IgG monoclonal antibody. Absorbance was read at 450 nm using a SpectraMax Paradigm reader (Molecular Devices), and IgG concentrations were calculated from a standard curve.

### 2.5 Immunization of humanized mice

Thirteen weeks after HSC transplantation into NOG‐EXL, the mice were immunized with inactivated SFTSV. The antigen was mixed with 2% Alhydrogel adjuvant (1:1; InvivoGen, vac‐alu‐250), yielding a final dose of 30 μg per mouse. Immunization was administered intraperitoneally twice at 2‐week intervals. Two weeks after the second immunization, the mice were euthanized using carbon dioxide (CO_2_), and the blood and spleens were collected. Serum was separated from the blood samples for analysis of virus‐specific neutralizing antibody titers.

### 2.6 Cells and viruses

Vero‐E6 cells were obtained from the Korean Cell Line Bank (21587) and maintained in Dulbecco's modified Eagle's medium (DMEM, 11995‐065; Gibco) supplemented with 10% FBS (10082‐147; Gibco) and 1% penicillin–streptomycin (P/S, 15140‐122; Gibco). All mammalian cells were grown at 37°C in a 5% CO₂ incubator. SFTSV KADGH/2013/Korea (GenBank accession no.: KU507553) was isolated from a patient infected with SFTSV in Korea.^18^ The SFTSV KADGH was propagated in Vero‐E6 cells using DMEM (10% FBS and 1% P/S). Virus stocks were grown in mammalian cells at 37°C, and the culture medium was harvested 5 days postinfection.

### 2.7 Virus titration

Vero‐E6 cells were infected with serial dilutions of SFTSV and incubated under an overlay of DMEM supplemented with 1.5% methylcellulose (M0387; Sigma), 10% FBS, and 1% P/S at 37°C for 2 days. The cell monolayers were then fixed with 4% paraformaldehyde for 10 min. After fixation, the cells were permeabilized using 0.5% Triton X‐100 (X100‐100 mL; Sigma) in PBS solution for 30 min at room temperature and washed once with 1× PBS. Permeabilized cells were incubated with a 1:500 dilution of anti‐SFTSV NP mouse antibody (in house) in a buffer supplemented with 1% bovine serum albumin (BSA, A9647‐100 g; Sigma) and 0.1% Triton X‐100 for 1 h, and then incubated with a 1:3000 dilution of HRP‐labeled anti‐mouse IgG (7076S; Cell Signaling Technology) for 1 h. Foci were visualized using TrueBlue peroxidase substrate solution (5510‐0030; SeraCare).

### 2.8 Virus inactivation

Harvested SFTSV samples were placed in a UVP Crosslinker (CL‐3000; Analytik Jena) and exposed to UV irradiation at 2500 mJ/cm^2^, and mixed thrice, totaling 10 000 mJ/cm^2^. Irradiated virus was collected in ultraclear centrifuge tubes (344058; Beckman) and centrifuged at 24 000 rpm for 2 h at 4°C using a swinging‐bucket rotor (SW28; Beckman). The supernatant was discarded, and the pellet was resuspended in 200 μL 1× PBS. Inactivated virus stocks were stored at −80°C.

### 2.9 Enzyme‐linked immunosorbent assay

Ninety‐six‐well plates (3690; Corning) were coated overnight at 4°C with recombinant SFTSV Gn (in house) and Gc glycoprotein (IT‐017‐005P; Immune Technology). The plates were blocked with 1% BSA in PBS for 1 h at room temperature, incubated with serially diluted mouse serum for 1 h, and washed with 0.05% Tween 20 in PBS (PBST). HRP‐conjugated antihuman IgG antibody (1:1000) in blocking buffer was added for 1 h and then washed with PBST; 100 μL of *o*‐phenylenediamine (P5412; Sigma) was added, and the reaction was stopped with 100 μL of 2 mol/L sulfuric acid. Absorbance was measured at 490 nm using a microplate reader (Thermo Fisher Scientific).

### 2.10 Focus reduction neutralization assay

Vero‐E6 cells were seeded at 1 × 10^5^ cells/mL in 24‐well plates. Humanized mouse serum was diluted (1:10) and serially diluted (1:4) in DMEM with 10% FBS and 1% P/S. Serum dilutions were mixed with SFTSV (80 FFU) and incubated on Vero‐E6 monolayers at 37°C for 1 h, with gentle mixing for 15 min. After incubation, the mixture was removed, and cells were washed thrice with PBS. Prewarmed overlay media (DMEM, 10% FBS, 1% P/S, 1.5% methylcellulose) was added, and plates were incubated at 37°C for 48 h. Cells were then washed with Dulbecco's PBS (14190‐144; Gibco), fixed with 4% paraformaldehyde (PC2031; Biosesang) for 10 min, and washed again. Anti‐SFTSV NP antibody (in house, 1:500) in 0.5% Triton X‐100/PBS was added for 1 h at room temperature with gentle mixing, and then the mixture was washed. HRP‐conjugated anti‐mouse IgG antibody in 0.5% Triton X‐100/PBS was added for 1 h at room temperature. SFTSV foci were visualized using True Blue HRP substrate and imaged using an ELISPOT reader (CTL). Each plate included two positive viral controls. FRNT_50_ value was calculated using GraphPad Prism.

### 2.11 Flow cytometry analysis

Peripheral blood and splenocytes were collected from humanized mice immunized with inactivated virus. Red blood cells were lysed using 10× RBC lysis buffer (420301; BioLegend, r7757‐100 mL, Sigma). T‐ and B‐cell subsets were analyzed using flow cytometry (FACSLyric or Aria III, BD Biosciences) after having stained with fluorescence‐conjugated antibodies. Peripheral blood cells were stained with antihuman CD45–APC‐Cy7 (557833) and CD3–V500‐C (652896). B cell profiling used CD19–BV711 (563036), CD20–Alexa Fluor 700 (560631), CD27–BV421 (742731), CD38–APC (567144), CD138–PE (552026), IgM–BV605 (562977), IgD–PE‐Cy7 (561314), and CD24–BV786 (568225) (all from BD Biosciences). Splenocytes were stained with CD3–BV421 (562877) and CD4–BV421 (317434) for T cells, and CD20–PerCP/Cy5.5 (302336) and CD27–PerCP/Cy5.5 (124214) for B cells (all from BioLegend). Human IgG^+^ B cells were detected using anti‐human IgG–FITC (555786, BD Pharmingen). Antibodies were incubated in 2% FBS buffer for 30 min on ice, protected from light. Data were analyzed using FlowJo software.

### 2.12 Statistical analysis

Statistical analyses were performed using GraphPad Prism. Differences between groups were evaluated using one‐way analysis of variance (ANOVA) and then post hoc Tukey's test, and a *p*‐value < 0.05 was considered statistically significant.

## 3 RESULTS

### 3.1 Humanized NOG‐EXL mice promoted immune cell engraftment and human IgG expression

**FIGURE 1:**
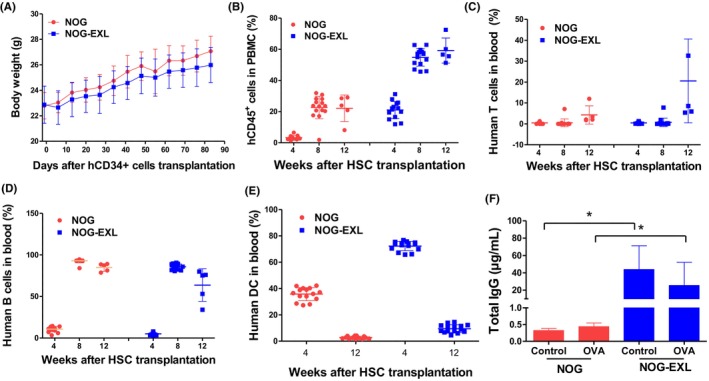
Comparison of human immune population in the HSC (hematopoietic stem cell)–transplanted NOG and NOG‐EXL mouse. (A) Body weight of humanized NOG‐EXL mice monitored weekly after CD34^+^ HSC transplantation. All mice exhibited gradual weight gain over time, with no significant differences among experimental groups. (B–D) Flow cytometric analysis of human immune cell subsets in the peripheral blood and spleen 12 weeks after HSC transplantation and 2 weeks postimmunization with inactivated SFTSV (severe fever with thrombocytopenia syndrome virus). (B) Proportion of human CD45^+^ leukocytes in the peripheral blood and spleen. (C) Proportion of human CD3^+^ T cells among CD45^+^ cells. (D) Proportion of human CD19^+^ B cells among CD45^+^ cells. (E) Proportion of human CD11b^+^ CD11c^+^ DCs (dendritic cells) among CD45^+^ cells. (F) Measurement of human IgG (immunoglobulin G) titers in serum using ELISA (enzyme‐linked immunosorbent assay) after immunization (*p < 0.05). Data are presented as mean ± SD (standard deviation). Each data point represents an individual mouse.

To investigate the differences in human hematopoiesis between humanized NOG‐EXL and humanized NOG mice, human CD34^+^ HSCs were transplanted into NOG‐EXL and NOG mice. After HSC transplantation, body weight changes were observed in both groups, which exhibited a gradual weight gain with no significant differences (Figure 1A). The percentage of hCD45^+^ cells in the blood of humanized NOG‐EXL mice increased significantly over time and remained higher than that of humanized NOG mice (Figure 1B). The proportion of human T cells gradually increased in humanized NOG‐EXL mice but remained low in the NOG group (Figure 1C). In humanized NOG‐EXL mice, the proportion of human B cells sharply increased 4 weeks after HSC transplantation, reaching a significantly higher level 12 weeks after HSC transplantation (Figure 1D). The proportion of human DCs increased 4 weeks after HSC transplantation in humanized NOG‐EXL mice and remained higher than that in humanized NOG mice at 12 weeks (Figure 1E). Humanized NOG‐EXL mice exhibited significantly higher total IgG levels than humanized NOG mice, regardless of OVA treatment (*p* < 0.05) (Figure 1F). Therefore, humanized NOG‐EXL mice were superior to conventional humanized NOG mice in terms of human immune cell reconstitution and response. Moreover, humanized NOG‐EXL mice exhibited higher human IgG production against foreign antigens.

### 3.2 Human immune cell reconstitution in HSC‐transplanted humanized NOG‐EXL mice before immunization

**FIGURE 2:**
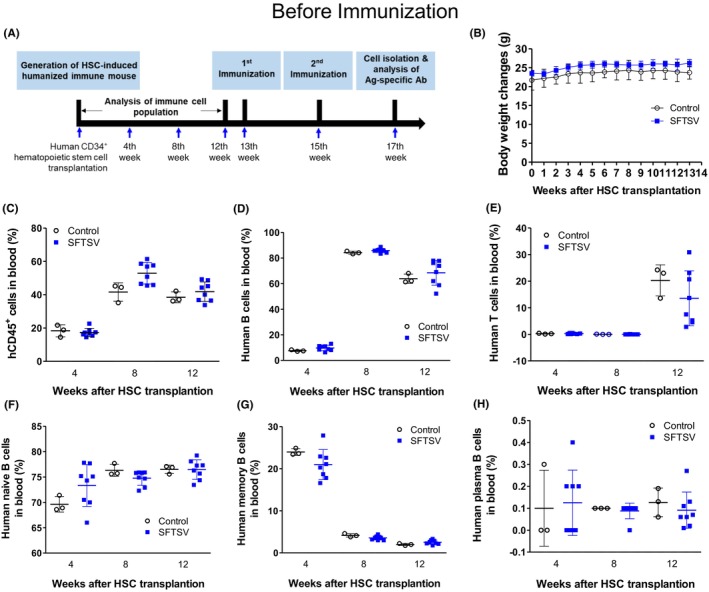
Immune cell population in HSC (hematopoietic stem cell)–transplanted NOG‐EXL mouse before inactivation virus immunization. (A) Schematic timeline of the experimental procedure. (B) Longitudinal monitoring of body weight changes in humanized mice over 14 weeks posttransplantation. (C–E) Flow cytometric analysis of human immune cell subsets in peripheral blood at indicated time points post‐HSC transplantation. (C) Percentage of human CD45^+^ leukocytes. (D) Percentage of human CD19^+^ B cells among CD45^+^ cells. (E) Percentage of human CD3^+^ T cells among CD45^+^ cells. (F–H) Subclassification of B‐cell populations in peripheral blood. (F) Naive B cells (CD27^−^IgD^+^). (G) Memory B cells (CD27^+^IgD^−^). (H) Plasma B cell (CD38^+^CD138^+^) frequencies were low in all groups prior to immunization. Data are presented as mean ± SD (standard deviation). Each point represents an individual mouse. Groups: Control (open circles) and inactivated SFTSV (severe fever with thrombocytopenia syndrome virus) (blue squares).

We characterized the immune cell population in the blood of humanized NOG‐EXL mice prior to immunization with inactivated SFTSV. Human CD34^+^ HSCs were transplanted into NOG‐EXL mice to produce humanized mice. Peripheral blood was sampled at 4, 8, and 12 weeks posttransplantation for immune cell profiling, and mice were immunized with the inactivated virus at 13 and 15 weeks, performing the final analyses at 17 weeks (Figure 2A). All groups exhibited gradual weight gain over time, with no significant differences (Figure 2B). The population of human CD45, T (CD3^+^), B (CD19^+^), and dendritic (CD11c^+^) cells in peripheral blood was analyzed every 4 weeks for 12 weeks after HSC engraftment. Immune cell populations were categorized per experimental groups after immunization to compare postimmunization immune responses (Figure 2). The merged data before group allocation for immunization are shown in Figure S1.

At 4 weeks after HSC transplantation, the proportions of hCD45^+^ cells were 18.3% ± 3.6% and 17.3% ± 2.5% in the control and SFTSV groups, respectively (Figure 2C). The proportions of human B cells (CD19^+^) were 7.6% ± 0.6%, 84.2% ± 1.1%, and 63.8% ± 3.65% in the control and 9.7% ± 2.1%, 85.9% ± 1.6%, and 68.5% ± 9.8% in the SFTSV groups at 4, 8, and 12 weeks after HSC transplantation, respectively (Figure 2D). Human T cells were hardly observed in all groups at 4 and 8 weeks after HSC transplantation, and at 12 weeks, the proportions were 20.3% ± 5.8% and 13.6% ± 10.3% in the control and SFTSV groups, respectively (Figure 2E). Naive B cells accounted for 69.6% ± 1.5%, 76.3% ± 1.2%, and 76.5% ± 0.8% in the control and 73.3% ± 4.1%, 74.8% ± 1.4%, and 76.5% ± 1.9% in the SFTSV groups at 4, 8, and 12 weeks after HSC transplantation, respectively (Figure 2F). Memory B cells accounted for 24.0% ± 0.8%, 4.2% ± 0.4%, and 2.0% ± 0.2% in the control and 21.0% ± 3.6%, 3.6% ± 0.5%, and 2.5% ± 0.5% in the SFTSV groups at 4, 8, and 12 weeks after HSC transplantation, respectively (Figure 2G). Plasma B cells were minimal at this time point across all the groups (Figure 2H).

### 3.3 Distribution of human immune cells in peripheral blood and spleen of HSC‐transplanted humanized mice after inactivated pathogen exposure

**FIGURE 3:**
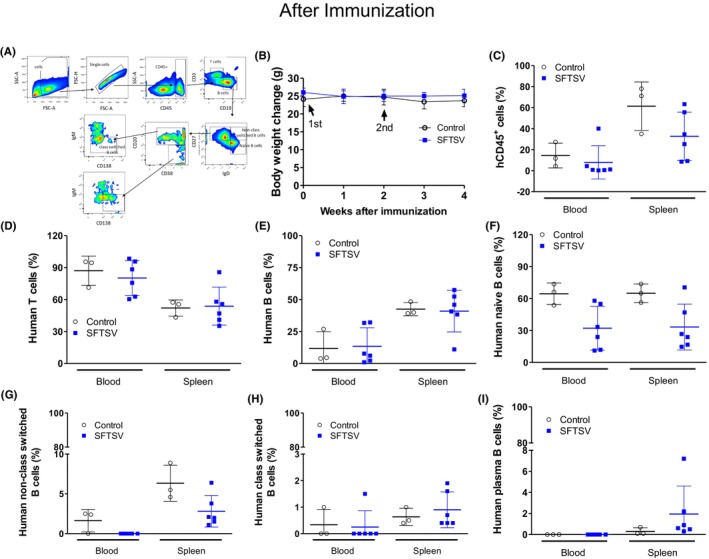
Immune cell population in HSC (hematopoietic stem cell)–transplanted NOG‐EXL mouse after inactivation virus immunization. (A) Representative flow cytometry gating strategy for identification of human immune cell subsets in humanized NOG‐EXL mice. (B) Body weights of humanized mice were monitored throughout the experiment. (C) Frequency of human CD45^+^ cells in the peripheral blood and spleen across experimental groups. (D, E) Proportions of human CD3^+^ T and CD19^+^ B cells among CD45^+^ cells in the peripheral blood and spleen. (F–I) Proportions of human naive, nonclass‐switched, class‐switched, and plasma B cells in the blood and spleen. Data represent mean ± SD (standard deviation). Each point corresponds to an individual mouse. Open circles: Control group; blue squares: SFTSV (severe fever with thrombocytopenia syndrome virus)–immunized group.

To evaluate the human immune cell responses elicited by exposure to inactivated virus in humanized mice, the distribution and composition of human immune cell subsets in the peripheral blood and spleen were analyzed. A gating strategy was performed for the discrimination of T, B, and plasma cells in mouse spleens (Figure 3A). Cells were first gated on lymphocytes using SSC‐A versus FSC‐A, followed by singlet discrimination (FSC‐H vs. FSC‐A). Human leukocytes were identified as CD45^+^ and subdivided into T (CD3^+^) and B (CD19^+^) cells. B cells were further classified into naive (CD27^−^IgD^+^), class‐switched (CD27^+^IgD^−^IgM^−^CD20^+^), nonclass‐switched (CD27^+^IgD^+^), and plasma (CD27^+^IgD^−^IgM^−^CD38^+^) B cells. This approach enabled the accurate quantification and phenotypic analysis of various B cell subpopulations. No statistically significant differences were observed in the body weights of control and inactivated SFTSV‐exposed groups (Figure 3B). The proportions of blood and spleen human CD45^+^ cells were 14.4% ± 11.7% and 61.4% ± 23.1% in the control and 7.9% ± 15.8% and 32.8% ± 23.0% in the SFTSV groups, respectively (Figure 3C). CD45^+^ human leukocytes were gated and further analyzed for lineage‐specific populations, including T (CD3^+^) and B (CD19^+^) cells, and antibody‐producing B cell subsets. The proportions of blood and spleen human T cells were 87.1% ± 13.8% and 52.1% ± 7.7% in the control and 80.2% ± 16.4% and 53.8% ± 17.8% in the SFTSV groups, respectively (Figure 3D). The proportions of human B cells in the blood and spleen were 11.8% ± 13.1% and 42.5% ± 5.1% in the control and 13.5% ± 14.5% and 41.0% ± 16.3% in the SFTSV groups, respectively (Figure 3E). Naive B cells accounted for 64.5% ± 10.1% and 64.9% ± 8.8% in the control and 32.2% ± 20.6% and 33.2% ± 21.6% in the SFTSV groups, respectively (Figure 3F). To assess humoral immune activation after inactivated pathogen administration, the distribution of B cell subtypes in the peripheral blood and spleen of humanized mice was analyzed, in which the proportions of nonclass‐switched B cells were 1.6% ± 1.4% and 6.3% ± 2.3% in the control and 0% and 2.8% ± 2.0% in the SFTSV groups, respectively (Figure 3G). Class‐switched B cells accounted for 0.3% ± 0.6% and 0.5% ± 0.3% in the control and 0.3% ± 0.6% and 0.9% ± 0.7% in the SFTSV groups, respectively (Figure 3H). Plasma B cells were scarce, accounting for ~0.0%–1.1% in the blood and 0.3% ± 0.3% (control) and 2.0% ± 2.7% (SFTSV) in the spleen (Figure 3I). These results suggest that inactivated virus exposure induced the functional maturation of human B cells in humanized mice, characterized by an increased production of class‐switched memory B and plasma cells, particularly within lymphoid organs, such as the spleen.

### 3.4 Humanized mouse model produced SFTSV‐specific human antibodies

**FIGURE 4:**
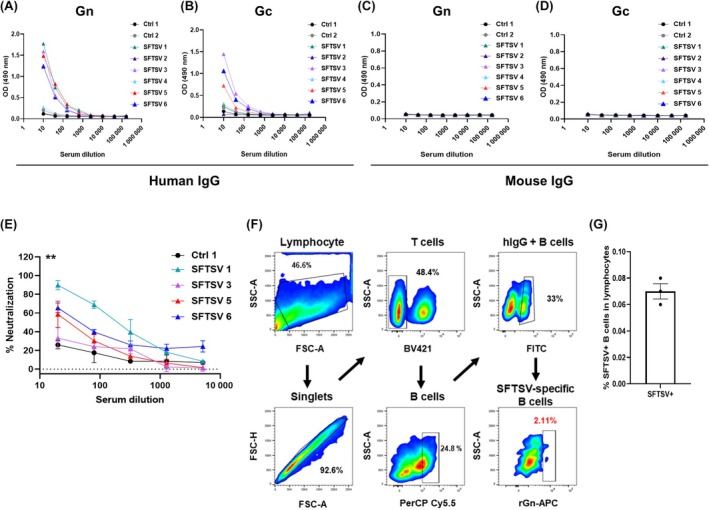
Humanized mouse model produces SFTSV (severe fever with thrombocytopenia syndrome virus)‐specific human antibodies. Immunization with inactivated SFTSV promoted the development of virus‐specific human antibodies in a humanized mouse model. Human IgG (immunoglobulin G) levels were assessed using enzyme‐linked immunosorbent assay (ELISA) with serum from humanized mice. Human antibodies against SFTSV (A) Gn and (B) Gc were detected using humanized mouse serum and horseradish peroxidase (HRP)–conjugated antihuman IgG antibodies. Virus‐specific mouse antibodies against SFTSV (C) Gn and (D) Gc were detected using humanized mouse serum and an anti‐mouse IgG‐HRP antibody. (E) The neutralizing activity of serum from humanized mice against SFTSV was determined using FRNT (focus reduction neutralization assay). All humanized serum samples were diluted to 1:20 in 1% BSA (bovine serum albumin) (A–D) or DMEM (Dulbecco's modified Eagle's medium) (E). Neutralization data are presented as mean ± standard error of the mean (SEM), with two replicates per experiment. Statistical comparisons were performed using a paired *t*‐test, as appropriate. **p < 0.01. (F) Flow cytometry gating scheme to identify human IgG‐expressing B cells in the splenocytes of SFTSV‐immunized humanized mice. Splenocytes can serve as subgates for isolating single lymphocytes. T and B cells, which are lymphocyte subsets, were also identified. B cells were selected from T cell‐negative populations by gating cells negative for T cell markers CD3^+^ and CD4^+^, and were identified within these populations using CD20^+^ and CD27^+^ markers. Next, IgG‐expressing B cells were selected using anti‐human IgG‐FITC (fluorescein isothiocyanate), and these cells were further identified as virus‐specific human B cells using a probe with a recombinant Gn protein conjugated to APC. Each B cell can be subgated to allow the identification of human IgG‐expressing B cells. Identified human IgG‐positive B cells are presented as percentages (%). (G) The proportion of SFTSV‐specific B cells among total lymphocytes is shown for each sorting experiment.

The SFTSV Gn and Gc mediate viral entry, and antibodies against Gn potently neutralize the virus and confer protection from infection, with Gn inducing a higher neutralizing antibody response than Gc.^19^ Therefore, we aimed to identify Gn‐specific antibodies. To confirm whether human IgG was present in humanized mouse serum, ELISA was conducted on separate plates coated with recombinant Gn and Gc. The production of antigen‐specific human antibodies was verified using secondary antibodies specific to human or mouse IgG (Figure 4A–D). Human IgG in humanized mouse serum, but not mouse IgG, bound to Gn and Gc in a dose‐dependent manner. The optical density (OD) values for the human IgG groups (OD_490_ = 1.2–1.7) showed an ~30‐fold increase compared with those of the mouse IgG groups (on average OD_490_ < 0.057), indicating that virus‐specific antibodies were produced in the humanized mouse model, with levels about six‐ to ninefold higher than those of the control groups (average OD_490_ < 0.19). Furthermore, their functional activity, particularly their neutralizing potential, was assessed. FRNT was performed using the serum from humanized mice immunized with the inactivated virus, which contained high levels of human IgG (Figure 4E; Figure S2). SFTSV‐immunized humanized mice exhibited neutralizing potency in a dose‐dependent manner; therefore, a humanized mouse model can produce neutralizing human IgG after immunization with inactivated viruses. At peak response, FRNT_50_ values in the SFTSV groups increased compared with the control group (FRNT_50_≈0.8), with 207 in SFTSV 1 (~259‐fold), 5 in SFTSV 3 (~6‐fold), 30 in SFTSV 5 (~38‐fold), and 48 in SFTSV 6 (~60‐fold). To determine whether virus‐specific antibodies could be selected from the spleen of a humanized mouse model, FACS and a gating strategy were used to isolate B cells expressing virus‐specific antibodies. To identify binding to the Gn protein, recombinant protein probes labeled with streptavidin‐APC were produced (Figure 4F). Lymphocytes were identified based on forward and side scattering; T cells were isolated from singlet CD3^+^ and CD4^+^ populations, whereas B cells, excluding T cells, were selected as CD20^+^ and CD27^+^ populations. To identify IgG‐expressing B cells, an IgG^+^ population was selected from the identified B cells. Sorting of specific B cells based on the gating strategy revealed that 2.11% of SFTSV Gn‐specific B cells were isolated from splenocytes of the humanized mouse model. Through three rounds of sorting, SFTSV^+^ B cells were identified at frequencies of 0.08% (12 of 15 536), 0.07% (8 of 10 873), and 0.06% (7 of 10 771) among total lymphocytes, respectively (Figure 4G; Figure S3A,B). Monoclonal antibodies specific for SFTSV, exhibiting weak binding, were subsequently derived from the sorted B cell populations (Figure S3C). Therefore, our data demonstrate that immunization of humanized NOG‐EXL mice caused the production of virus‐specific B cells.

## 4 DISCUSSION

Humanized mouse models have significantly advanced our understanding of human immune responses, particularly in the context of infectious diseases.^5^, ^6^, ^7^, ^8^ Although previous studies using HSC‐transplanted humanized mouse models have demonstrated the production of human antibodies, a few have confirmed the production of antigen‐specific antibodies, and no studies have shown that humanized NOG‐EXL mice produce antigen‐specific human antibodies, let alone their isolation, expression, or functional characterization. These limitations are likely attributable to incomplete B‐cell maturation and suboptimal humoral immune responses in humanized mouse models. Among these models, the NOG‐EXL mice, expressing the human cytokines IL‐3 and GM‐CSF, showed promise in enhancing the engraftment and differentiation of human hematopoietic cells. Our study aimed to evaluate the ability of HSC‐transplanted humanized NOG‐EXL mice to produce antigen‐specific human antibodies after immunization with an inactivated virus. We found that NOG‐EXL mice support the robust engraftment of human immune cells, including T, B, and DCs, leading to the production of antigen‐specific human IgG antibodies. This agrees with previous reports indicating that expressing human IL‐3 and GM‐CSF in immunodeficient mice enhances myeloid and lymphoid lineage development.^15^ The ability of these mice to produce class‐switched, antigen‐specific antibodies underscores their potential as a platform for studying human humoral immune responses. Although we evaluated the production of antigen‐specific IgG antibodies, it remained unclear whether these responses were sustained over time or conferred protective immunity against subsequent pathogen exposure. Additionally, the extent to which immune responses in NOG‐EXL mice recapitulate those in humans warrants further investigation. Another consideration is the potential of the NOG‐EXL model to support the development of germinal center reactions, crucial for producing high‐affinity, long‐lived plasma and memory B cells. Studies suggest that humanized mouse models exhibit limited germinal center formation due to the absence of human follicular DCs and other supporting stromal elements. Consistent with this, we interpret the high percentage of memory B cells at week 4 as a transient engraftment and expansion of preexisting mature B cells under lymphopenic conditions, followed by progressive replenishment of the compartment with HSC‐derived naive B cells. Future studies incorporating human lymphoid tissues or engineered microenvironments may enhance the accuracy of these models.

Immunization with inactivated SFTSV resulted in the production of only human antibodies in humanized NOG‐EXL mice, suggesting that this model responds to defined antigens and is suitable for inducing antigen‐specific human antibodies. The absence of mouse IgG indicates a deficiency in the mouse immune system. Therefore, the potential development of graft‐versus‐host disease in humanized mice should be carefully considered. The human antibodies produced in this model exhibited neutralizing activity against SFTSV, highlighting their potential as platforms for developing therapeutic human antibodies.

Using FACS, virus‐specific B cells were successfully sorted from spleen cells of humanized mice. The isolation of antigen‐reactive cells implies that their corresponding antibody gene sequences can be characterized, validating the potential of this method for therapeutic antibody development. To obtain the complementarity‐determining regions sequences of human IgG from these B cells, phage display,^20^ and single B cell‐based antibody discovery methods can be applied.^21^ A screening is currently in progress to identify antibodies with potent neutralizing activity using these antibody sequences. Furthermore, we aimed to enhance the efficacy of the identified neutralizing antibodies by applying bispecific antibody designs and Fc engineering strategies.^22^, ^23^

To evaluate whether several viruses, including newly emerging pathogens, can be adapted to this humanized mouse platform, mice were immunized with inactivated SFTSV. Immunization induced virus‐specific human IgG, demonstrating the functionality of the platform. Particularly, humanized NOG‐EXL mice produced SFTSV‐neutralizing anti‐Gn human IgGs. In our study, humanized NOG‐EXL mice produced higher levels of virus‐specific human antibodies than humanized NOG mice. This likely reflects the intrinsic properties of the NOG‐EXL strain, in which human GM‐CSF and IL‐3 expression enhances human myeloid reconstitution and antigen presentation, leading to more efficient antiviral humoral responses. These advantages render NOG‐EXL mice more beneficial than conventional NOG mice for the efficient production of human antibodies against viral antigens.

These findings suggest that the immunization of this mouse model with viral antigens could enable the production of virus‐specific antibodies, supporting their application in therapeutic antibody development. Overall, this study strongly supports the potential of the established humanized mouse model across a broad spectrum of viruses. However, the artificial immune environment in the humanized mouse model demonstrated quantitative limitations in human antibody production. Therefore, strategies that regulate class switch^24^ and promote the proliferation and differentiation of immune cells to enhance antibody production are needed.^25^ Additionally, optimizing immune conditions, such as the type of adjuvant and antigen, is required to increase antibody production in humanized mice.

## 5 CONCLUSION

Our study supports the HSC‐transplanted humanized NOG‐EXL mice as producers of antigen‐specific human antibodies. Nevertheless, further refinement is necessary to fully replicate the complexity of human immune responses and enhance the translational relevance of humanized mouse models in immunological research. Future studies should focus on producing therapeutic antibodies for treating viral infectious diseases. In this context, the humanized mouse platform developed in this study may facilitate an effective response to emerging viral pandemics.

## AUTHOR CONTRIBUTIONS

**Dong Hoon Lee:** Investigation; writing – original draft; writing – review and editing. **Jiyeong Bae:** Investigation; writing – original draft; writing – review and editing. **Sumi Kim:** Investigation. **Chan Young Song:** Formal analysis. **Jung Hyu Shin:** Formal analysis. **Eun Hee Kim:** Formal analysis. **Chan Ho Jang:** Formal analysis. **Young‐sun Yun:** Writing – review and editing. **Dong‐sook Lee:** Writing – review and editing. **Hyuk Chu:** Writing – review and editing. **Jang‐Hoon Choi:** Project administration; supervision; writing – review and editing. **Chan Woo Kim:** Project administration; supervision; writing – review and editing.

## FUNDING INFORMATION

This work was supported by a research program funded by the Korea Centers for Disease Control and Prevention (grant numbers: 2022‐ER1701‐00, 2024‐ER1702‐00, 2022‐NI‐041‐02, and 2025‐NI‐014‐00).

## CONFLICT OF INTEREST STATEMENT

The authors declare no conflicts of interest.

## ETHICS STATEMENT

All procedures involving animals were approved by the IACUC of OSONG Medical Innovation Foundation (KBIOHealth) and performed following ethical guidelines (KBIO‐IACUC‐2022‐164‐1 and KBIO‐IACUC‐2024‐139).

## Supporting information


Figure S1.

Figure S2.

Figure S3.
