# Supplementary figures and images for: A humanized NOG‐EXL mouse model for producing severe fever with thrombocytopenia syndrome virus–reactive human antibodies
Source: Animal Model Exp Med. 2026 Feb 26;9(2):378–88. doi: 10.1002/ame2.70140 (PMC13042686; doi:10.1002/ame2.70140)

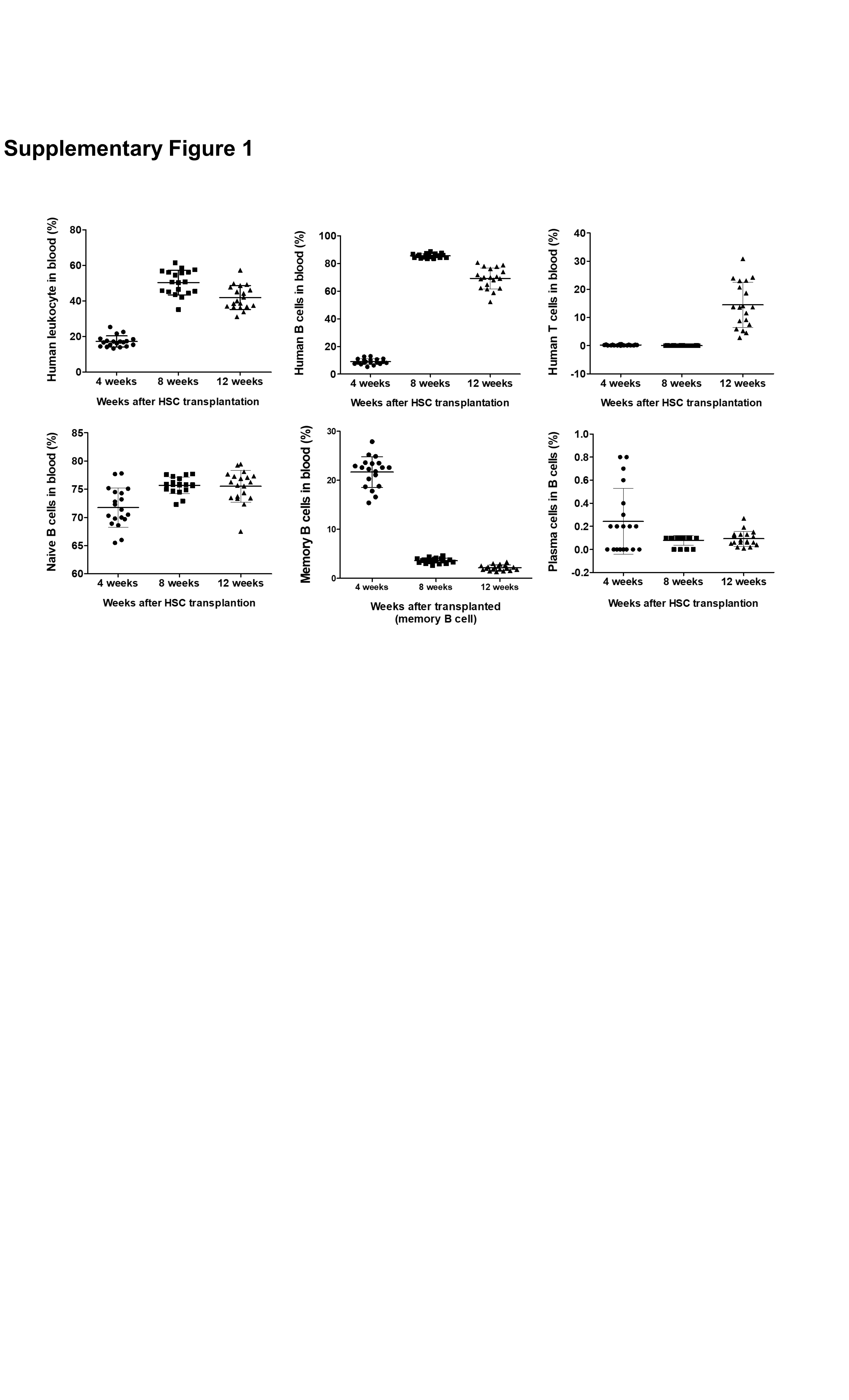

Supplement: Supplementary file 1 — Figure S1. [file AME2-9-378-s003.tif]

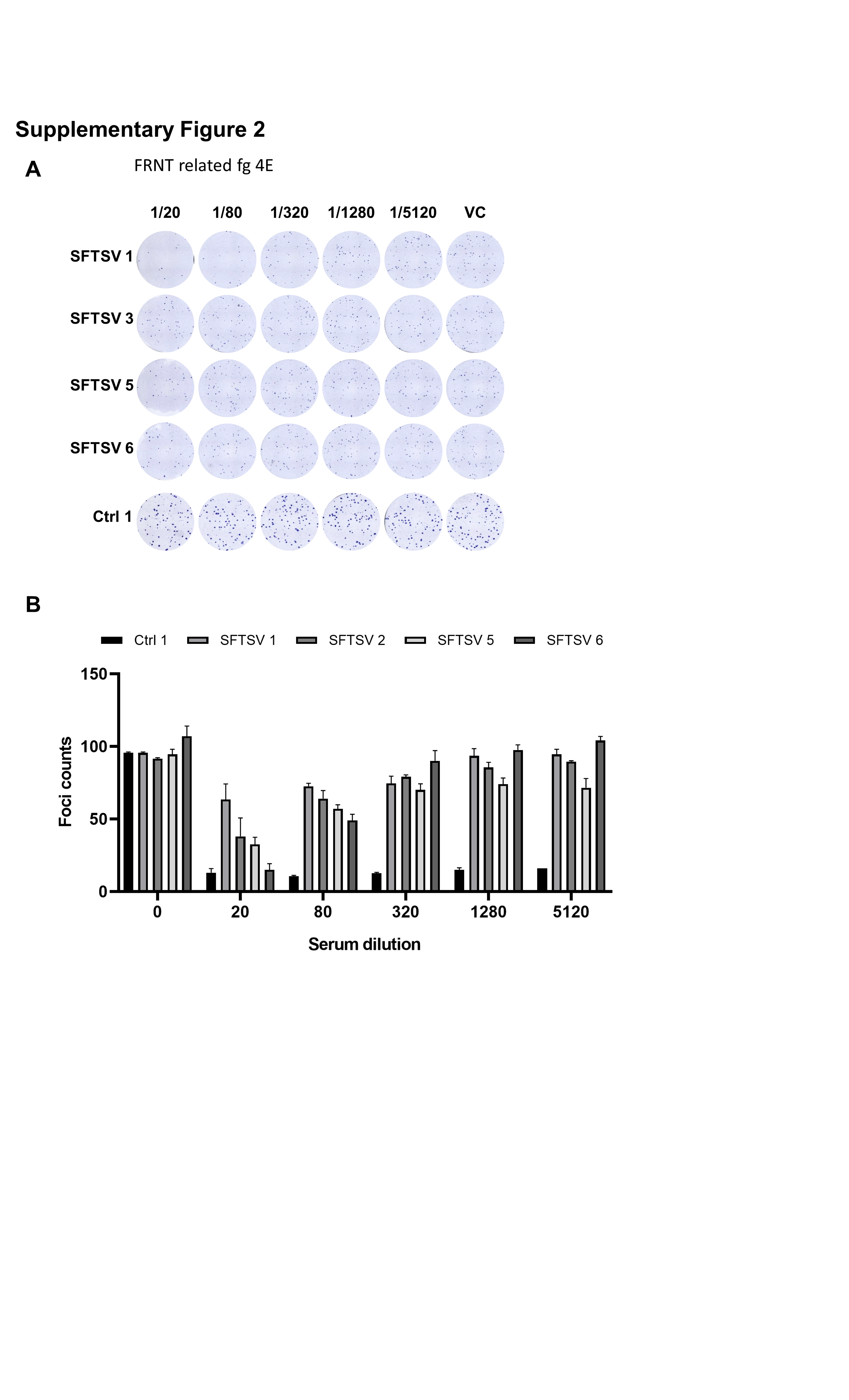

Supplement: Supplementary file 2 — Figure S2. [file AME2-9-378-s001.tif]

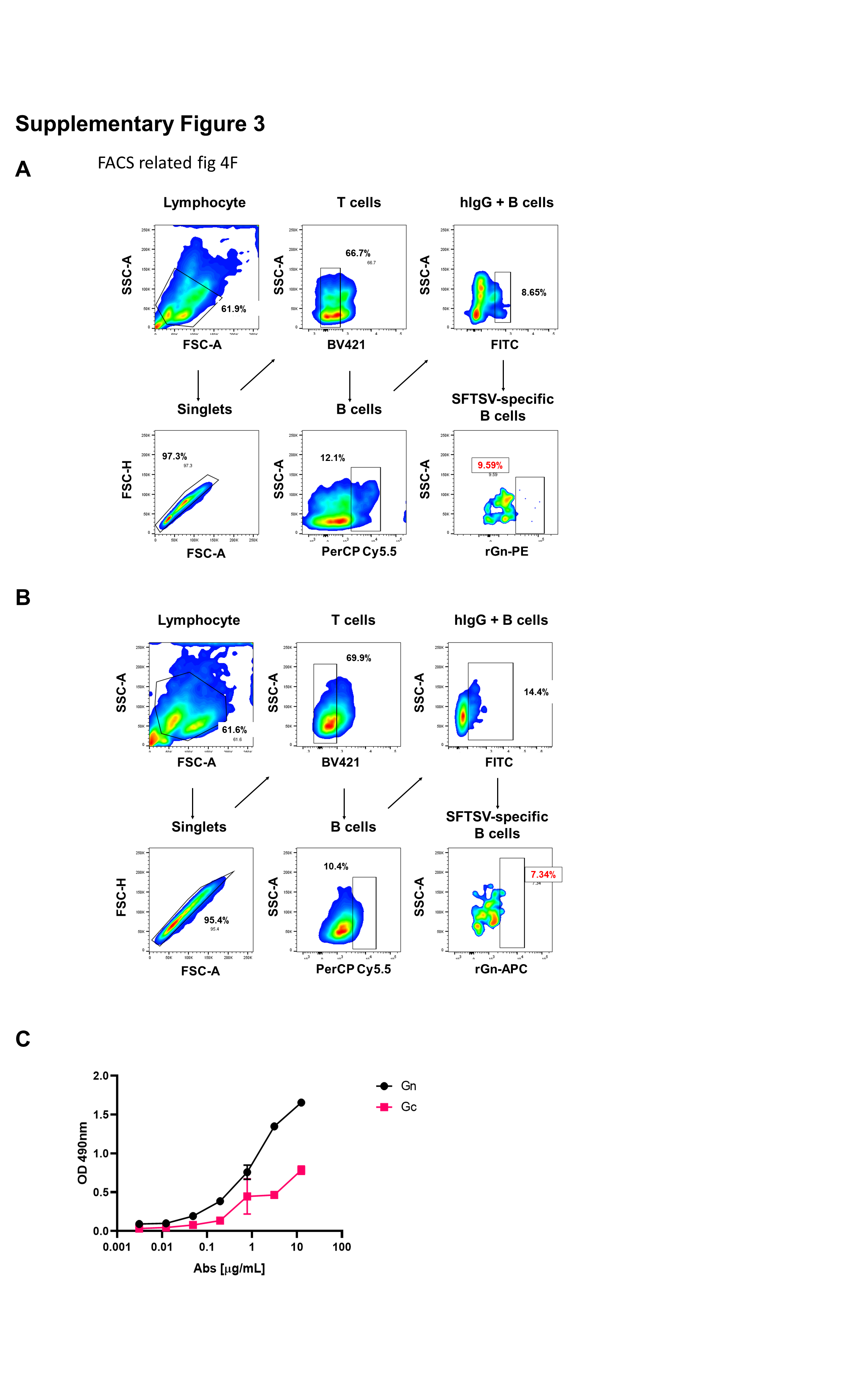

Supplement: Supplementary file 3 — Figure S3. [file AME2-9-378-s002.tif]
